# Transmembrane and coiled-coil domains 3 is a diagnostic biomarker for predicting immune checkpoint blockade efficacy in hepatocellular carcinoma

**DOI:** 10.3389/fgene.2022.1006357

**Published:** 2022-09-29

**Authors:** Xinyao Hu, Hua Zhu, Shi Feng, Chaoqun Wang, Yingze Ye, Xiaoxing Xiong

**Affiliations:** ^1^ Cancer Center, Renmin Hospital of Wuhan University, Wuhan University, Wuhan, China; ^2^ Department of Neurosurgery, The Affiliated Huzhou Hospital, Zhejiang University School of Medicine (Huzhou Central Hospital), Huzhou, China; ^3^ Department of Neurosurgery, Renmin Hospital of Wuhan University, Wuhan University, Wuhan, China

**Keywords:** TMCO3, ICB, diagnostics, immune infiltrates, LIHC

## Abstract

Liver hepatocellular carcinoma (LIHC) is a malignancy with a high mortality and morbidity rate worldwide. However, the pathogenesis of LIHC has still not been thoroughly studied. Transmembrane and coiled-coil domains 3 (TMCO3) encodes a monovalent cation, a member of the proton transducer 2 (CPA2) family of transporter proteins. In the present study, TMCO3 expression and its relationship with cancer prognosis, as well as its immunological role in LIHC were studied by bioinformatic analysis. We found the significant overexpression of TMCO3 in LIHC in the TCGA, HCCDB, and GEO databases. In LIHC patients, high TMCO3 expression was related to poorer overall survival (OS) and TMCO3 had good predictive accuracy for prognosis. Moreover, TMCO3 was linked to the infiltrates of certain immune cells in LIHC. The correlation of TMCO3 with immune checkpoints was also revealed. Moreover, patients with LIHC with low TMCO3 expression showed a better response to immune checkpoint blockade (ICB) than those with LIHC with high TMCO3 expression. GO and KEGG enrichment analyses indicated that TMCO3 was probably involved in the microtubule cytoskeleton organization involved in mitosis, small GTPase mediated signal transduction, and TGF-β pathway. In conclusion, TMCO3 may be a potential biomarker for LIHC prognosis and immunotherapy.

## Introduction

Liver hepatocellular carcinoma (LIHC) is the most common subtype of primary liver cancer, usually diagnosed at a late stage, and has become the second deadliest type of cancer worldwide (Sung et al., 2021). It is estimated that around one million people die from liver cancer each year (Villanueva, 2019; Llovet et al., 2021). The high rate of death from LIHC is due to its late diagnosis and high incidence of liver dysfunction. For patients with advanced diagnosis, postoperative metastasis and recurrence, the 5-years survival rate is poor, less than 50% (Anwanwan et al., 2020). Currently, treatment options for LIHC are limited, including surgery, interventional therapy, chemotherapy and immunotherapy (Anwanwan et al., 2020). Nevertheless, the 5-years survival rate of patients is not significantly improved due to factors such as post-operative recurrence, early metastasis, and the development of drug resistance (Morise et al., 2014). There is a lack of effective methods to predict patient prognosis and provide personalized treatment. Therefore, there is an urgent need to find more credible diagnostic biomarkers and to develop novel indicators to forecast patient survival and thus deliver personalized treatment therapies.

Transmembrane and coiled-coil domains 3 (TMCO3) encodes proton antiporter 2 (CPA2) family of transporter proteins, a member of the monovalent cation. This family members usually combine the output of a monovalent cation with the input of a proton that crosses the cell membrane. It has been reported that this gene is mutated in patients with a rare genetic visual defect: corneal drop cataract (Chen et al., 2016). However, the role of this gene in LIHC or other human cancers has not been studied.

Tumor microenvironment (TME) is a critical factor influencing the progression and treatment of LIHC (Kurebayashi et al., 2018). There is growing evidence that tumor-infiltrating immune cells (TIICs) influence the biological behavior of LIHC cells and eventually influence patient prognosis (Zheng et al., 2017; Li et al., 2020). Additionally, several recent studies and clinical trials have shown that immunotherapy or combination immunotherapy has the potential to improve the prognosis of patients with advanced LIHC (Rizzo et al., 2021; Rizzo et al., 2022a). In patients with unresectable untreated LIHC, disease-free survival (DFS) and overall survival (OS) are significantly longer with combination ICB, and there is greater hope that patients will undergo subsequent surgery (Rizzo and Brandi, 2021; Rizzo et al., 2022b). Our previous study has identified several biomarkers that predict the response to ICB treatment in LIHC patients, including TUBA1B (Hu et al., 2022a), TUBA1C (Hu et al., 2022b), and KIFC1 (Li et al., 2021). However, whether TMCO3 promotes the progression of LIHC or influences the immune infiltration of LIHC, or may be a predictor of ICB treatment, has not been reported.

In our study, we firstly demonstrated that TMCO3 expression was upregulated in LIHC and was correlated with poor prognosis by analyzing the data from the TCGA, GEO, HPA, and HCCDB database. Secondly, through functional enrichment analysis, we found that TMCO3 was related to multiple tumor-related signaling pathways. Patients with LIHC with different TMCO3 expression showed different outcomes to ICB treatment. In conclusion, our results suggest that TMCO3 could be used as a new prognostic biomarker and a possible therapeutic target for LIHC.

## Methods

### Data acquisition and TMCO3 expression analysis

The data for TMCO3 expression analysis was obtained from the TCGA (https://tcga.xenahubs.net) (accessed on 06 June 2022) and GEO (GES112790, including 183 LIHC samples and 15 normal samples) (http://www.ncbi.nlm.nih.gov/geo/) (accessed on 09 June 2022) databases. The “Wilcox.test” method was used to assess the differential TMCO3 mRNA expression in LIHC and normal tissues. We applied “Kruskal-wallis test” to explore the expression TMCO3 in different stages of LIHC. Boxplot was drawn using the “ggpubr” R package. The TIMER (https://cistrome.shinyapps.io/timer/) (accessed on 17 June 2022), HCCDB (http://lifeome.net/database/hccdb.html) (accessed on 11 June 2022) and GEPIA (http://gepia.cancer-pku.cn/index.html) (accessed on 20 June 2022) databases were employed to explore the expression of TMCO3 as we previously done (Hu et al., 2022a).

Immunohistochemical (IHC) images of the TMCO3 protein in normal and LIHC tissues were downloaded to evaluate the differential TMCO3 protein expression in the human protein atlas (HPA, https://www.proteinatlas.org/) database (accessed on 18 June 2022). In addition, the location of TMCO3 in U-2 OS, A-131, and U251 MG cell lines were assessed in the HPA database.

### Univariate and multivariate Cox regression analyses

We used univariate and multivariate Cox regression analyses (*p* < 0.05 as significant) to assess the effect of TMCO3 expression and other clinical features (including: age, sex, race, pTNM-stage and grade) on OS. To screen whether TMCO3 and these clinicopathologic factors could be regarded as independent contributors for LIHC, we developed a nomogram model. We performed univariate and multivariate Cox hazard regression analyses on LIHC samples from the TCGA database using the R package “forestplot”. Furthermore, to predict potential OS in patients with LIHC, we used the R ‘rms’ package and the “survivor” package to build a validated nomogram model. Once each element was divided into points, we summed the points for each parameter to calculate the total number of points. Lastly, we validated the nomogram using the harmonic index (c-index) and calibration curves.

### Analysis of the association of TMCO3 with survival of LIHC patients

The relevance of TMCO3 to the OS and DFS of LIHC was explored in the GEPIA database. Additionally, the relationship of TMCO3 with other human cancers was also assessed in this database (accessed on 25 June 2022).

### Immune infiltration analysis

We used the TIMER database (accessed on 30 June 2022) to explore the correlation of TMCO3 expression with several immune cells infiltration in LIHC. In addition, the CIBERSORT method which was developed to evaluate the abundance of particular cells in hybrid cell populations using gene expression datasets was also employed to evaluate the correlation of TMCO3 expression with other immune cell infiltrates as previously reported (Hu et al., 2021; Zhu et al., 2021). Through using R packages “ggplot2,” “ggpubr,” and “ggExtra”, we assessed the correlation of TMCO3 with immune filtration.

### Immune checkpoints analysis

Subsequently, the expression of several immune checkpoints, including CTLA4, PDCD1, SIGLEC15, HAVCR2, TIGIT, CD274, LAG3, and PDCD1LG2 was extracted in the high and low TMCO3 expression group (median as the cut-off). The two-gene correlation map was implemented by the R package “ggstatsplot”. Spearman’s correlation analysis was performed to characterize associations between quantitative variables that were not normally distributed. The Tumor Immune Dysfunction and Exclusion (TIDE) algorithm was applied to assess patients’ response to ICB treatment.

### Analysis of differentially expressed genes

We divided the obtained expression data into low and high expression groups based on median TMCO3 expression levels, which were then further analyzed by unpaired Student’s t-test in the ‘DESeq2’ R package. |log2 fold change (FC)|>1 and adjusted *p* < 0.05 were taken as the thresholds for DEGs.

### Functional enrichment

We selected the enriched KEGG signaling pathway analysis to illustrate the main biological roles of the major potential mRNAs. Gene ontology (GO) analysis was performed on potential mRNAs targets. We clustered the biological processes (BPs) of potential targets using the “ClusterProfiler” package in R software. Additionally, the LinkedOmics (http://www.linkedomics.org/) (accessed on 29 June 2022) was employed to perform the GSEA analysis (including GO and KEGG enrichment analyses) of TMCO3 in LIHC (Yu et al., 2022).

### Protein Protein interaction (PPI) network

The TMCO3 PPI information were built from STRING (https://cn.string-db.org/) (accessed on 1 July 2022) website to further study the role of TMCO3 in LIHC.

## Results

### The expression of TMCO3 in LIHC

**FIGURE 1:**
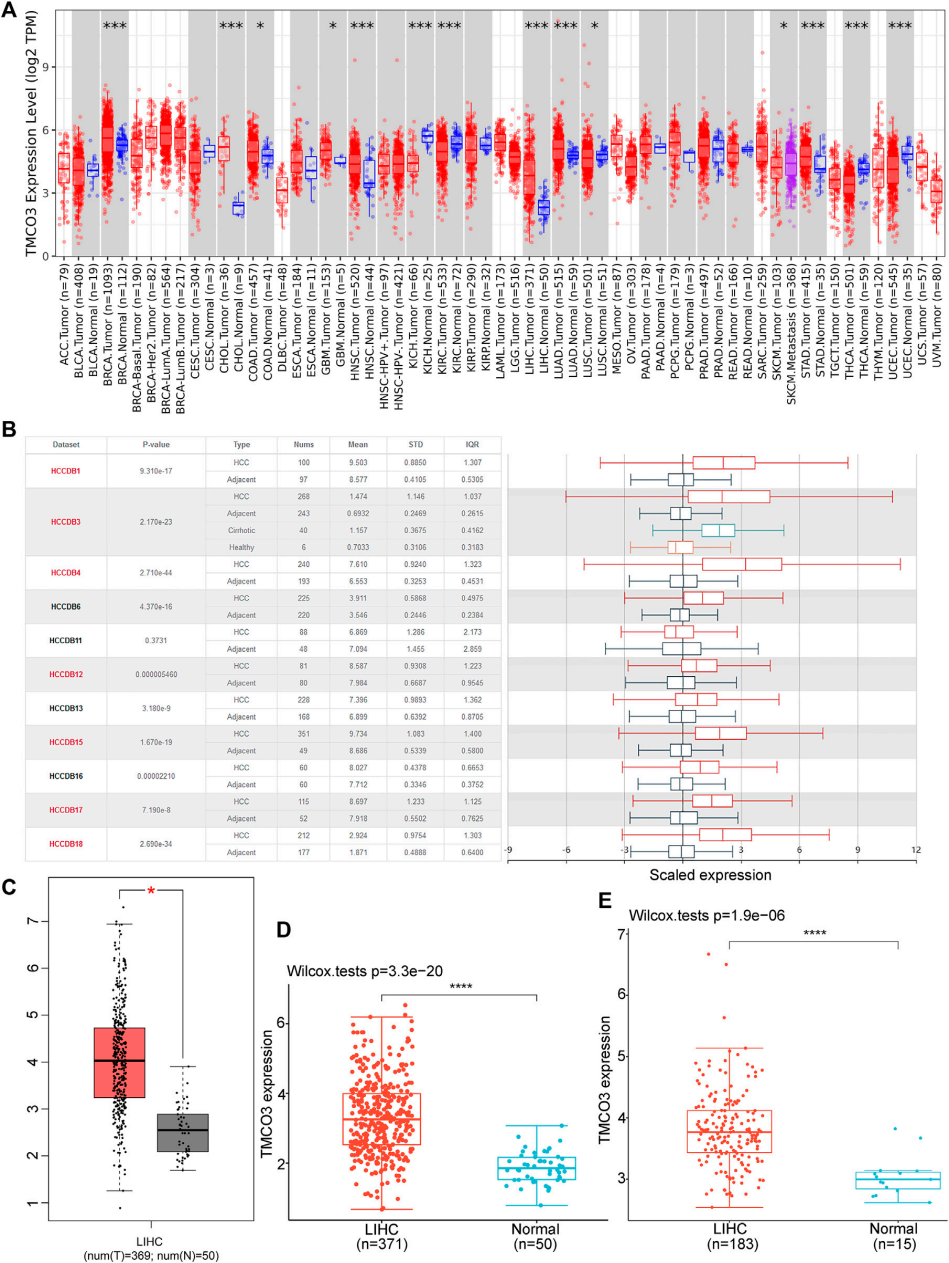
The TMCO3 expression in normal tissues and LIHC **(A)** The TMCO3 mRNA expression in human cancers and normal tissues. **(B)** The TMCO3 expression in LIHC and adjacent tissues in HCCDB database. The TMCO3 expression in LIHC and normal tissues in GEPIA **(C)**, TCGA **(D)**, and GEO **(E)** databases. *p < 0.05, ***p < 0.001, ****p < 0.0001.

**FIGURE 2:**
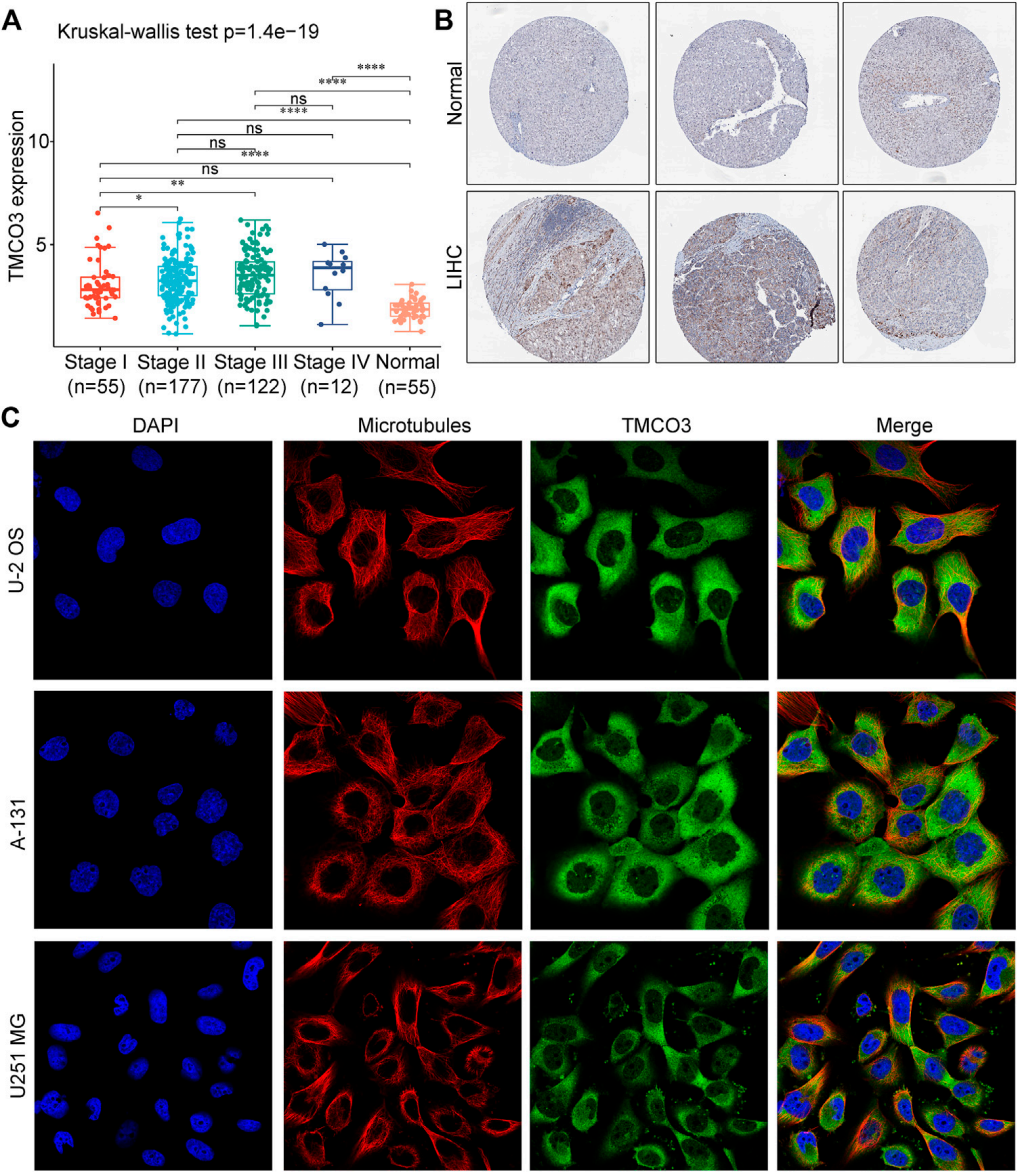
The expression and location of TMCO3 protein **(A)** The TMCO3 protein IHC in LIHC and normal tissues from HPA database. **(B)** The association with TMCO3 expression and tumor stages of LIHC. **(C)** The immunofluorescence staining of TMCO3 and microtubules in U-2 OS, A-131, and U251 MG cell lines in HPA database. *p < 0.05, **p < 0.01, ****p < 0.0001, ns: no significant difference.

We obtained TMCO3 mRNA expression levels from the TCGA database and investigated them to identify differential expression patterns between tumor and normal tissues and found that in several tumor tissues, the expression of TMCO3 was higher than in the respective normal adjacent tissues, including ESCA, COAD, GBM, HNSC, LIHC, and LUAD (Figure 1A). The data in HCCDB also confirmed the elevated TMCO3 expression in LIHC than in normal liver tissue (Figure 1B). The same results were obtained for the TCGA-based data (Figures 1C,D). Additionally, by analyzing the data downloaded from GEO, we also found that TMCO3 was up-regulated in LIHC than normal tissues (Figure 1E). We further assessed the expression of TMCO3 in different stages of LIHC, and we observed that TMCO3 expression was higher in stage II than in stage I but lower than in stage III (Figure 2A).

Next, we investigated the TMCO3 protein expression in LIHC and normal liver tissues in HPA database. The IHC staining of TMCO3 was stronger in LIHC than in normal tissues (Figure 2B). Moreover, in U-2 OS, A-131, and U251 MG cell lines, the proteins of TMCO3 were mainly localized in the cytoplasm (Figure 2C).

### The prognostic value of TMCO3 in LIHC

**FIGURE 3:**
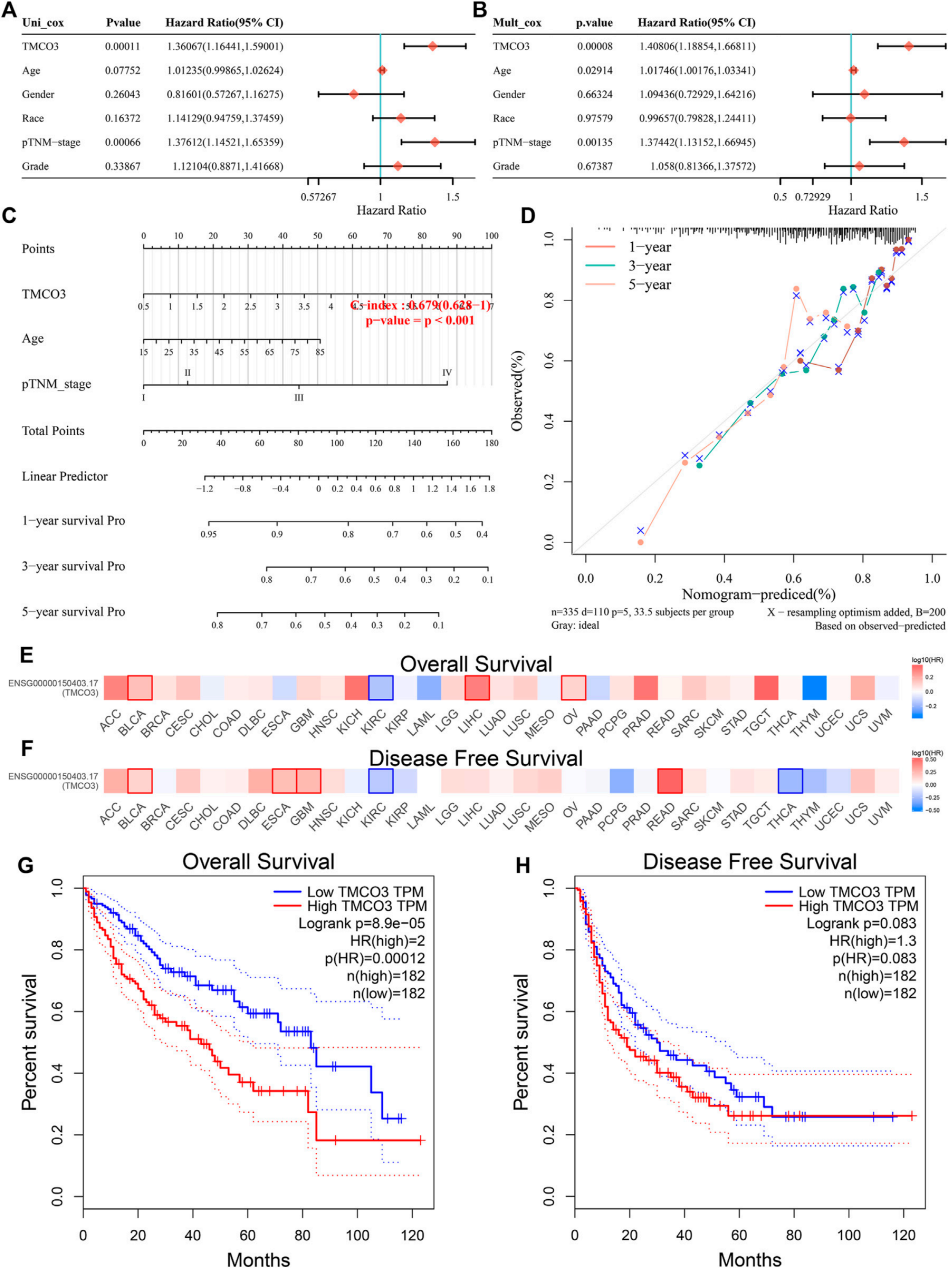
The prognostic value of TMCO3 in LIHC **(A)** Univariate and **(B)** multifactorial Cox analysis of TMCO3 and other clinical factors in LIHC **(C)** The nomogram and **(D)** Calibration curves of TMCO3, age, and pTNM-stage was established to predict 1-, 3-, and 5-years OS in LIHC patients. The association of TMCO3 with the OS **(E)** and DFS **(F)** in pan-cancer. The correlation of TMCO3 with OS **(G)** and DFS **(H)** in LIHC.

The univariate and multivariate Cox regression analyses revealed that TMCO3 expression may be an independent prognostic factor in LIHC (*p* < 0.001) (Figures 3A,B). The nomogram model demonstrated that TMCO3 can be an independent factor associated with OS and has an accurate predictive ability for 1-, 3-, and 5-years prognosis (Figures 3C,D). In GEPIA database, we observed that TMCO3 was related to the OS and RFS in several cancers, including BLCA and KIRC (Figures 3E,F). In LIHC, the expression of TMCO3 was correlated with poor OS than low TMCO3 expression (*p* = 8.9e−05) (Figure 3G). However, the TMCO3 expression was not correlated to DFS (*p* = 0.083) (Figure 3H).

### The correlation of TMCO3 with immune cell infiltrates in LIHC

**FIGURE 4:**
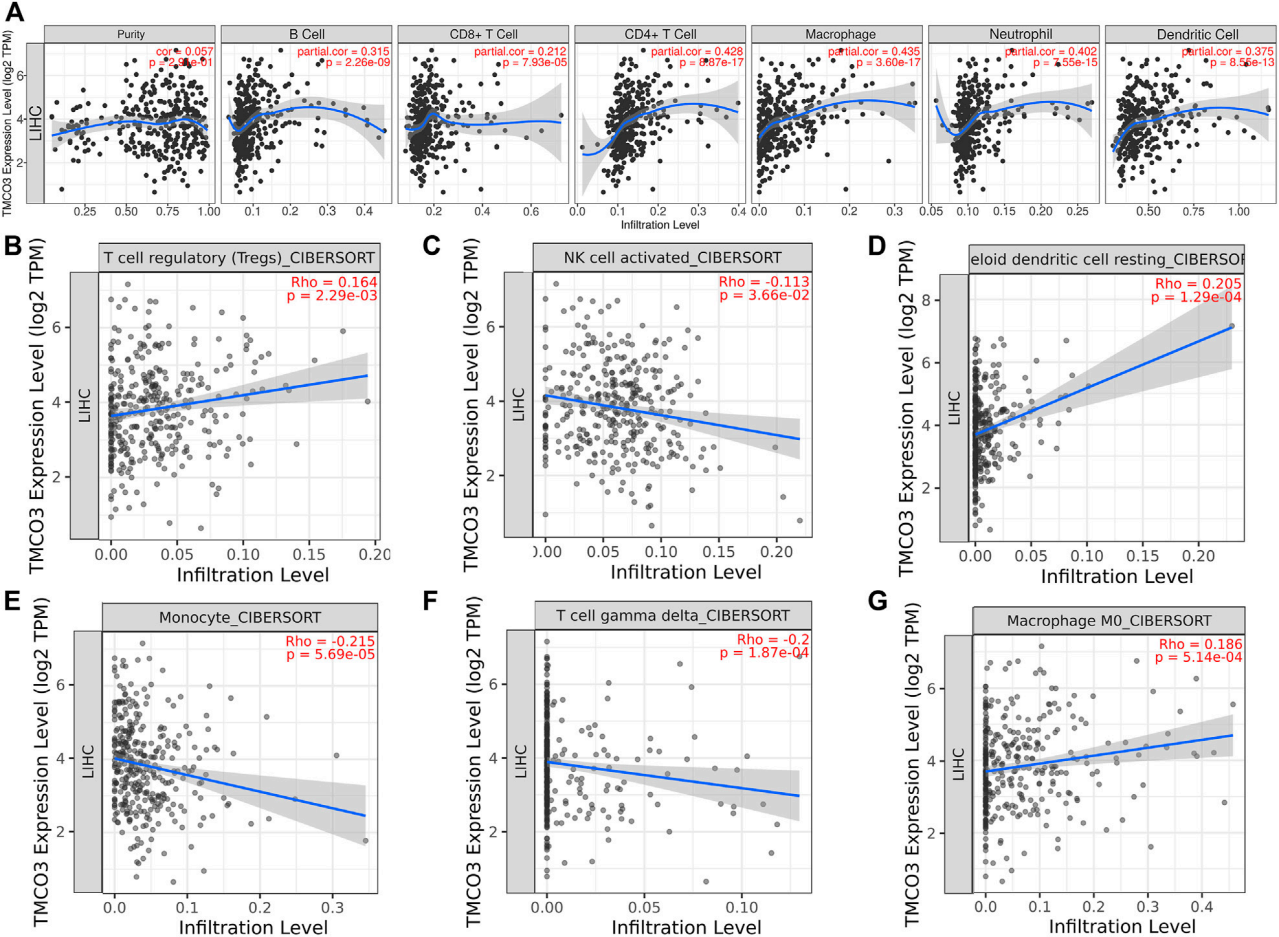
The association of TMCO3 with immune cell infiltrates **(A)** The association of TMCO3 with several immune-infiltrating cells in LIHC. The correlation of TMCO3 with Tregs **(B)**, activated NK cells **(C)**, resting myeloid dendritic cells **(D)**, monocytes **(E)**, gamma delta T cells **(F)**, and macrophages **(G)**.

We used the TIMER database to examine the relevance of TMCO3 to immune cell infiltration in LIHC, and we found that TMCO3 expression was associated with the infiltration of B cells (Cor = 0.315, *p* = 2.26e−09), CD4^+^ T cells (Cor = 0.428, *p* = 8.87e−17), CD8^+^ T cells (Cor = 0.212, *p* = 7.93e−05), dendritic cells (Cor = 0.375, *p* = 8.55e−13), neutrophil (Cor = 0.402, *p* = 8.55e−13), and macrophages (Cor = 0.435, *p* = 3.60e−17), (Figure 4A). Additionally, we applied the CIBERSORT algorithm to assess the relevance of TMCO3 to the infiltration of other immune cells. We found that TMCO3 was related to the infiltration levels of Tregs (Cor = 0.164, *p* = 2.29e−03) (Figure 4B), activated NK cells (Cor = *c*0.113, *p* = 3.66e−02) (Figure 4C), resting myeloid dendritic cells (Cor = 0.205, *p* = 1.29e−04) (Figure 4D), monocytes (Cor = −0.215, *p* = 1.87e−04) (Figure 4E), gamma delta T cells (Cor = −0.2, *p* = 1.87e−04) (Figure 4F), and macrophage M0 (Cor = 0.186, *p* = 5.14e−04) (Figure 4G).

### TMCO3 was associated with immune checkpoints and LIHC patient response to ICB

**FIGURE 5:**
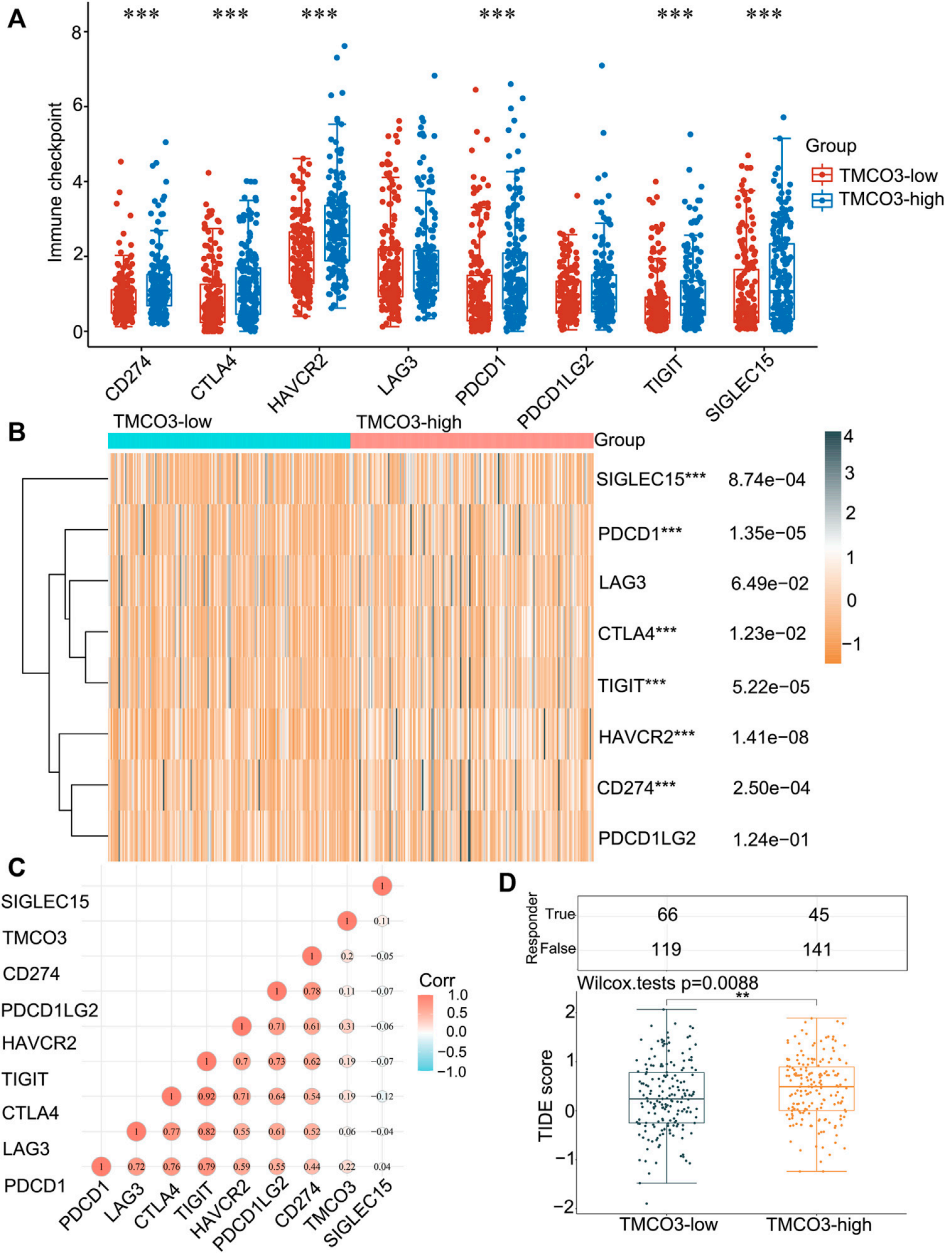
The correlation of TMCO3 with the immune checkpoints and response to ICB **(A,B)** The immune checkpoints expression in TMCO3-low and TMCO3-high groups in LIHC. **(C)** The correlation between TMCO3 and immune checkpoints in LIHC. **(D)** The TIDE scores in TMCO3-low and TMCO3-high groups in LIHC. **p < 0.01, ***p < 0.001.

Subsequently, we investigated the immune checkpoints expression in low and high TMCO3 expression group. The expression of CD274 (*p* = 8.74e−04), CTLA4 (*p* = 1.23e−02), HAVCR2 (*p* = 1.14e−08), PDCD1(*p* = 1.35e−05), TIGIT (*p* = 5.22e−05), SIGLEC15 (*p* = 8.74e−04) was higher in TMCO3-high group than in TMCO3-low group (Figures 5A,B). The TMCO3 expression was associated with the expression of PDCD1 (Cor = 0.22), CD274 (Cor = 0.2), HAVCR2 (Cor = 0.31), TIGIT (Cor = 0.19), and CTLA4 (Cor = 0.19) (Figure 5C). Moreover, patients with LIHC with high TMCO3 expression had higher TIDE scores than the group with low TMCO3 expression (Figure 5D). These results suggest that TMCO3 can be applied as a predictor for ICB efficacy in LIHC.

### The potential functions of TMCO3 in LIHC

**FIGURE 6:**
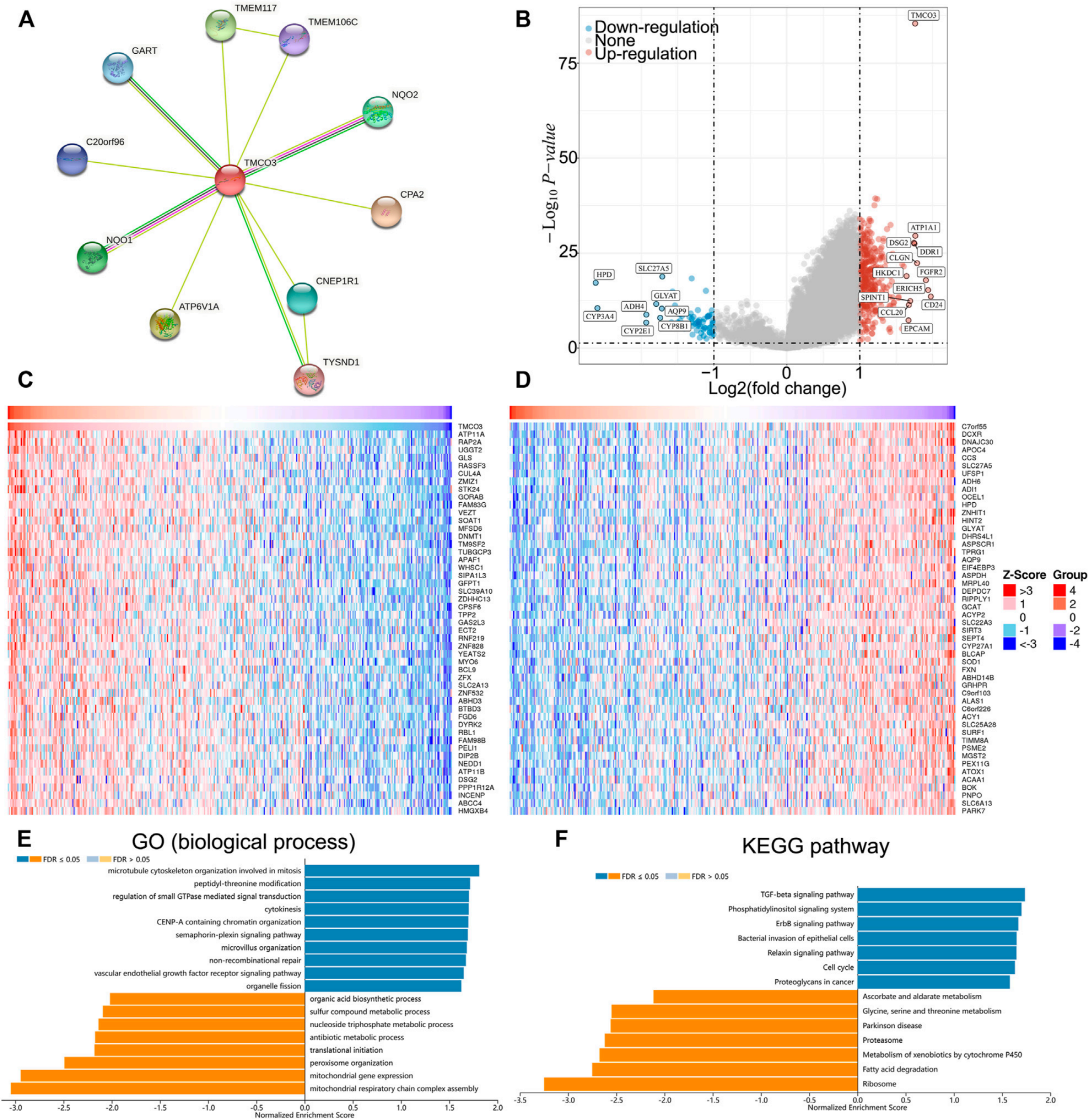
The potential role of TMCO3 in LIHC **(A)** PPI network of TMCO3 in LIHC. **(B)** The volcano plot showing the differential genes in TMCO3-high and TMCO3-low groups in LIHC. The top 50 genes that positively **(C)** and negatively **(D)** associated with TMCO3 in LIHC **(E)** Biological process GO analysis and KEGG pathway analysis **(F)** of TMCO3 in LIHC.

**FIGURE 7:**
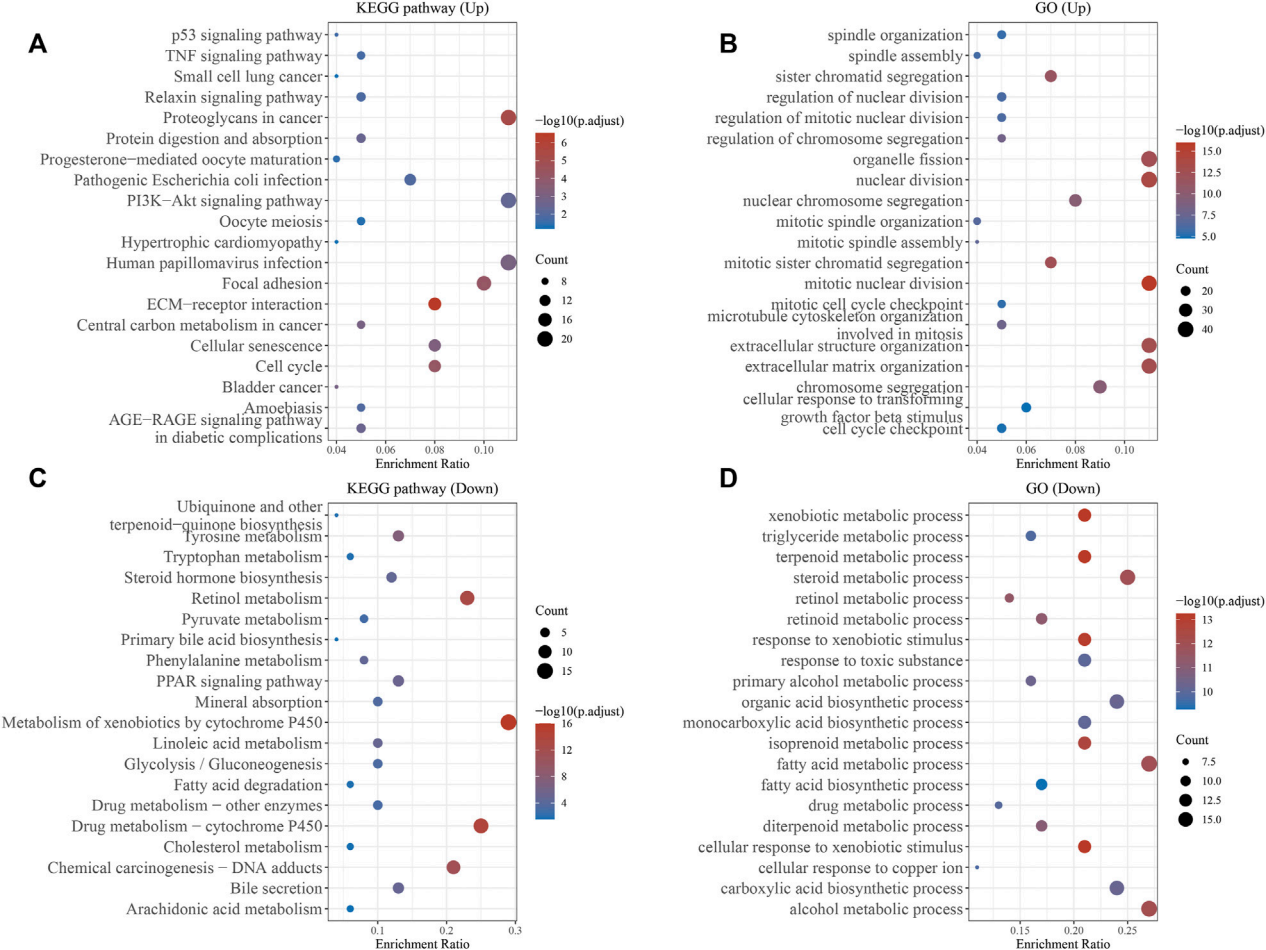
The enrichment analyses of differential genes. KEGG pathway analysis **(A)** and GO analysis **(B)** of the up-regulated genes. KEGG pathway analysis **(C)** and GO analysis **(D)** of the down-regulated genes.

We established the PPI network of TMCO3 to assess the potential proteins that interplay with TMCO3 by STRING. The results indicated that TMCO3 may interplay with GART, TEME117, NQO2, CPA2, TYSND1, ATP6V1A, NQO1, C20orf96, and CNEP1R1 (Figure 6A). The volcano plots of differential genes in low and high groups of TMCO3 expression were shown in Figure 6B. The top 50 genes positively or negatively associated with TMOC3 was shown in Figures 6C,D. The top ten genes that was positively associated TMCO3 included ATP11A, RAP2A, UGGT2, GLS, RASSF3, CUL4A, ZMIZ1, STK24, GORAB, and FAM83G (Figure 6C). The top ten genes that was negatively associated with TMCO3 included C7orf55, DCXR, DNAJC30, APOC4, CCS, SLC27A5, UFSP1, ADH6, ADI1, and OCEL1 (Figure 6D). The GSEA analysis based on GO analysis indicated that TMCO3 was involved in microtubule cytoskeleton, peptidyl-threonine modification, regulation of small GTPase mediated signal transduction, cytokinesis, CENP-A containing chromatin organization, semaphoring-plexin pathway, microvillus organization, mitochondrial respiratory chain complex assembly, mitochondrial gene expression, vascular endothelial growth factor receptor pathway, peroxisome organization, translational initiation, antibiotic metabolic process in LIHC (Figure 6E). The GSEA analysis based on KEGG pathway analysis revealed that TMCO3 was associated with the TGF-beta pathway, phosphatidylinositol signaling system, ErbB pathway, proteoglycans in cancer and cell cycle in LIHC (Figure 6F). The KEGG pathway enrichment results of differentially upregulated genes (TMCO3-high vs. TMCO3-low group) indicated that proteoglycans in cancer, PI3K-akt pathway, cell cycle and focal adhesion were enriched in these up-regulated genes (Figure 7A). GO analysis indicated that organelle fission, nuclear division, mitotic spindle organization, extracellular matrix organization, and extracellular structure organization were related to these genes (Figure 7B). The KEGG pathway enrichment results of differentially down-regulated genes (TMCO3-high vs. TMCO3-low group) demonstrated that retinol metabolism, drug metabolism-cytochrome P450, metabolism of xenobiotics by cytochrome P450, and cholesterol metabolism were enriched in these down-regulated genes (Figure 7C). GO analysis revealed that xenobiotic metabolic process, fatty acid metabolic process, steroid metabolic process, and alcohol metabolic process were enriched in these down-regulated genes (Figure 7D).

## Discussion

LIHC has a high morbidity and mortality rate and is the third leading cause of tumor-related deaths worldwide. The overall 5-years survival rate for patients with LIHC is less than 20% (Villanueva, 2019; Nault and Villanueva, 2021). In spite of many efforts in early diagnosis and new treatments, the outcome of patients with LIHC is still unsatisfactory owing to the specific TME and the tumor heterogeneity (Gao et al., 2015; Bruix et al., 2019). Studies on hepatocarcinogenesis, heterogeneity and drug resistance have drawn attention and efforts to TME. Tumors rely on TME to maintain their proliferation, metastasis and invasion (Quail and Joyce, 2013). Briefly, the TME comprises resident stromal cells, recruited immune cells, and non-cellular components capable of interacting with cancer cells. In addition, TIICs may be related to immune disruption as the tumor grows (Quail and Joyce, 2013). Many studies have elucidated immune targets, particularly ICBs (El Dika et al., 2019; Liu et al., 2019). However, ICBs can lead to complexity and heterogeneity of TME in LIHC and do not have the desired therapeutic effect on LIHC patients (El Dika et al., 2019; Zeng et al., 2020). Nevertheless, ICBs remain a new therapeutic advancement for patients with LIHC, particularly for those with advanced LIHC (Zeng et al., 2020). Multiple studies have demonstrated that immune infiltration, a hallmark of TME and tumor heterogeneity, responds better to ICB. The presence of genetic indicators of T helper cells and CD8 T cells contributes to a better outcome according to previous studies on several malignancies (Chen and Han, 2015). Our previous studies have revealed several biomarker that may be served as predictors for LIHC and predict the therapeutic insensitivity to ICB, including TUBA1C (Hu et al., 2022b), KIFC1 (Li et al., 2021), TUBA1B (Hu et al., 2022a). Herein, a novel biomarker has been revealed that may be regarded to be a diagnostic and immunological predictor.

In our research, we identified that the protein and mRNA expression of TMCO3 was significantly higher in LIHC tissues than in normal liver tissues. Subsequently, we investigated the relevance of TMCO3 to the prognosis of LIHC and found that the OS of LIHC was poorer in the TMCO3 high expression group, suggesting that elevated TMCO3 expression predicted poor LIHC prognosis. In addition, the association of TMCO3 with the clinicopathology of LIHC was also confirmed. We found that TUBA1B was significantly higher in stage III than in stage I of LIHC. In addition, univariate and multifactorial Cox analyses showed that TMCO3 was an independent prognostic factor for LIHC. Next, we constructed a nomogram to predict 1-, 3-, and 5-years OS in patients with LIHC. In conclusion, TMCO3 is a potential prognostic biomarker for LIHC.

Tumor immune cell infiltration is correlated with tumor progression and response to immunotherapy (Binnewies et al., 2018; Zhu et al., 2020; Zhu et al., 2022). In our study, we observed a strong positive association between TMCO3 expression and infiltration of several immune cell types, suggesting a higher degree of tumor immune cell infiltration in LIHC patients with high TMCO3 expression. The top four immune cell types that showed a significant positive correlation with RPS3A expression were neutrophils, CD4 T cells, DCs and macrophages. Therefore, it is expected to increase tumor immune cell infiltration by targeting TMCO3. Furthermore, TMCO3 expression was positively related to the expression of most of the immune checkpoint molecules we observed in public database samples, suggesting that this gene may promote the synthesis or expression of immunosuppressive molecules through unknown mechanisms. More importantly, TIDE scores were elevated in the TMCO3 high expression group than in the low expression group, indicating that LIHC patients with lower TMCO3 expression has increased therapeutic insensitivity to ICB in LIHC. Therefore, TMCO3 can be a biomarker to predict the responsiveness of LIHC to ICB treatment.

Ultimately, we investigated the genes and pathways related to TMOC3 to explore the potential role of TMCO3. The results indicated that TMCO3 may interplay with GART, TEME117, NQO2, CPA2, TYSND1, ATP6V1A, NQO1, C20orf96, and CNEP1R1. The top ten genes that was positively associated TMCO3 included ATP11A, RAP2A, UGGT2, GLS, RASSF3, CUL4A, ZMIZ1, STK24, GORAB, and FAM83G. The top ten genes that was negatively associated with TMCO3 included C7orf55, DCXR, DNAJC30, APOC4, CCS, SLC27A5, UFSP1, ADH6, ADI1, and OCEL1. These proteins may interplay with TMCO3 to exert tumorigenic effects. The GO analysis indicated that TMCO3 was involved in microtubule cytoskeleton, peptidyl-threonine modification, regulation of small GTPase mediated signal transduction, cytokinesis, CENP-A containing chromatin organization, semaphoring-plexin pathway, vascular endothelial growth factor receptor pathway, microvillus organization, mitochondrial respiratory chain complex assembly, mitochondrial gene expression, peroxisome organization, translational initiation, antibiotic metabolic process in LIHC. The KEGG pathway analysis revealed that TMCO3 was related to the TGF-beta pathway, phosphatidylinositol signaling system, ErbB pathway, proteoglycans in cancer and cell cycle in LIHC. These results may indicate the potential role of TMCO3 in LIHC.

There are also several limitations in this work. Due to the lack of validation experiments in this study, in the future investigation, we will further verify the more accurate mechanism of action of TMCO3 in LIHC by *in vitro in vivo* experiments. In addition, the heterogeneity of tumors, the health status of patients, and changes in the immune microenvironment may cause immune checkpoint non-response and poor therapeutic effects. This is an important reason for the poor efficacy of many immunotherapies at present. Moreover, this work was conducted only based on the mRNA and protein expression profile. As the development of single-cell sequence technology, more and more novel advanced methods (such as Single-cell Multi-omics Gene co-Regulatory algorithm (Song et al., 2022), graph-based convolutional networks (Song and Su, 2021), BIOMEX (Taverna et al., 2020), and single-cell Graph Convolutional Network (Song et al., 2021)) are important in discovering potential targets, pathogenesis, and specific cells in tumors. Applying them to future research and data analysis to gain a deeper understanding of tumorigenesis and development is necessary.

## Conclusion

Taken together, we found for the first time that TMCO3 has a poor prognosis in hepatocellular carcinoma and explored its possible mechanisms in LIHC. We confirmed the correlation of TMCO3 with LIHC immune infiltration and suggested that TMCO3 may serve as a new immunotherapeutic biomarker. Patients with LIHC with high TMCO3 expression may be more sensitive to ICB therapy. Thus, our findings will help to further provide precise immunotherapy for LIHC patients.

## Data Availability

The original contributions presented in the study are included in the article/supplementary material, further inquiries can be directed to the corresponding authors.
