# Corrigendum: Transmembrane and coiled-coil domains 3 is a diagnostic biomarker for predicting immune checkpoint blockade efficacy in hepatocellular carcinoma

**DOI:** 10.3389/fgene.2022.1117731

**Published:** 2023-01-16

**Authors:** Xinyao Hu, Hua Zhu, Shi Feng, Chaoqun Wang, Yingze Ye, Xiaoxing Xiong

**Affiliations:** ^1^ Cancer Center, Renmin Hospital of Wuhan University, Wuhan University, Wuhan, China; ^2^ Department of Neurosurgery, The Affiliated Huzhou Hospital, Zhejiang University School of Medicine (Huzhou Central Hospital), Huzhou, China; ^3^ Department of Neurosurgery, Renmin Hospital of Wuhan University, Wuhan, China

**Keywords:** TMCO3, ICB, diagnostics, immune infiltrates, LIHC

In the published article, there was an error regarding the **Affiliation** for “Xiaoxing Xiong^2,3^.” As well as having affiliations 2,3, he should also have “1 Cancer Center, Renmin Hospital of Wuhan University, Wuhan University, Wuhan, China.”

The authors apologize for this error and state that this does not change the scientific conclusions of the article in any way. The original article has been updated.

